# The effects of Qingchang Ligan formula on hepatic encephalopathy in mouse model: results from gut microbiome-metabolomics analysis

**DOI:** 10.3389/fcimb.2024.1381209

**Published:** 2024-08-16

**Authors:** Ziwei Yang, Shuhui Liu, Feili Wei, Jianhua Hu

**Affiliations:** ^1^ Beijing Youan Hospital, Capital Medical University, Beijing, China; ^2^ Beijing Institute of Hepatology, Beijing Youan Hospital, Capital Medical University, Beijing, China

**Keywords:** gut microbiota, hepatic encephalopathy, traditional Chinese medicine, metabolomics, inflammation

## Abstract

**Background:**

Hepatic encephalopathy (HE) is a neurological disorder resulting from advanced liver injury. HE has a high mortality rate and poor prognosis. The pathogenesis of HE is still unclear, which has led to the lack of a satisfactory specific treatment method. There is increasing evidence that the intestinal flora affects the communication between the gut and the brain in the pathogenesis of HE. Adjusting the intestinal flora has had a beneficial effect on HE in recent studies, and the Qingchang Ligan formula (QCLG) has been shown in previous studies to regulate intestinal flora and metabolites. In this study, we established a thioacetamide-induced HE mouse model to evaluate the protective effect of QCLG on HE and explore its potential mechanism, which also demonstrated that intestinal flora dysbiosis is involved in the pathogenesis of HE.

**Methods:**

Mice were intraperitoneally injected with thioacetamide (TAA, 150 mg/kg) to induce HE. Additionally, they were orally administered Qingchang Ligan Formula (QCLG) at a dose of 6.725 g/kg·d for seven days, while control mice received an equal volume of saline via gavage. Subsequently, samples were subjected to 16S ribosomal ribonucleic acid (rRNA) gene sequencing, high-performance liquid chromatography-mass spectrometry (LC-MS), and RNA-sequencing (RNA-seq) analysis.

**Result:**

QCLG improved weight loss, cognitive impairment, neurological function scores, blood ammonia, and brain gene expression of interleukin-6 (TNF-α), Interleukin-1β (IL-1β), and interleukin-6 (IL-6) induced by HE. Moreover, QCLG increased the levels of liver function indicators, including alanine aminotransferase (ALT), aspartate aminotransferase (AST), and serum TNF-α, IL-1β, and IL-6. 16S RNA sequencing revealed increased *Oscillibacter, Colidextribacter*, and *Helicobacter* in TAA-induced mouse fecal samples. Also, the abundance of *Bifidobacterium* decreases TAA-induced mouse fecal samples. In contrast, QCLG treatment significantly restored the gut microbial community. Metabolomics indicated significant differences in some metabolites among the normal control, treatment, and model groups, including 5-methoxytryptophan, Daidzein, Stercobilin, and Plumieride (PLU).

**Conclusion:**

QCLG can alleviate neuroinflammation and prevent HE caused by liver injury by regulating intestinal flora in mouse models.

## Introduction

Hepatic encephalopathy (HE), also known as hepatic coma, refers to a syndrome of central nervous system dysfunction resulting from metabolic disorders caused by severe liver disease. HE is a common and serious complication of chronic liver disease and acute liver failure (Bloom et al., 2022). Primary research directions for understanding HE pathogenesis involve theories such as ammonia poisoning, pseudo neurotransmitter hypothesis, intestinal flora, inflammatory response, and more (Baishuang et al., 2021; Feng and Weiqun, 2023).

Research on the microbiota-gut-brain (MGB) has advanced significantly in recent decades. A growing body of evidence indicates the involvement of microbial communities in the development of neurological diseases. Disruption of gut microbiota may trigger low-level inflammation, including neuroinflammation. Intestinal microflora is involved in the formation of nerves, the immune system, or other basic processes in the process of growth. During the development of HE, intestinal dysbiosis can not only induce a chronic inflammatory state in the intestinal epithelium but also increase neuroinflammation through the microbiota-gut-brain axis. Persistent inflammation in the gastrointestinal tract associated with dysbiosis can lead to the destruction of intestinal barrier integrity and increased permeability. Subsequently, pro-inflammatory microbial products such as lipopolysaccharide (LPS) and cytokines will cross the damaged barrier and enter the blood circulation, causing systemic inflammation. Subsequently, these pro-inflammatory molecules in systemic circulation may induce the destruction of the blood-brain barrier (BBB) (Zhao et al., 2021; Won et al., 2022). Studies have reported an increased number of pathogenic bacteria in the intestinal tract of HE patients compared to normal individuals, with an enhanced synergy among harmful bacteria (Elsaid and Rustgi, 2020). Studies have shown improvement in patients treated with Fecal Microbiota Transplantation (FMT) (Bloom et al., 2021; Li et al., 2022). Utilizing subjects with higher probiotic abundance, specifically *Lachnospiraceae* and *Ruminococcaceae*, as donors for FMT treatment in HE patients has demonstrated effective improvement in cognitive dysfunction associated with HE (Afecto et al., 2021).

Additionally, a study assessed 127 HE patients through cognitive testing. Notably, the FMT cohort showed improved cognitive performance and maintained this improvement over long-term follow-up (Tun et al., 2022). Overall, these reports suggest a pivotal role of alterations in intestinal microbiota in the pathogenesis of HE. However, the underlying mechanism requires further exploration.

Currently, primary clinical treatment options consist of lactulose and rifaximin, both of which exhibit obvious drawbacks (Jindal and Jagdish, 2019); given the limited treatment options for HE, discovering safe and effective drugs is highly beneficial. Qingchang Ligan Formula (QCLG) is an intrahospital prescription at Beijing You’an Hospital and has been clinically used there for many years. QCLG can reduce inflammation levels, ameliorate liver damage by regulating intestinal flora and reducing alanine and aspartate aminotransferase (ALT and AST) (Yin et al., 2022). Traditional Chinese medicine formulations are usually composed of various components, which thus highlights their feature of possessing multiple components and targets. Consequently, the impact of QCLG may extend beyond the diseases that have previously been identified. Against the background of the above information, our study aimed to investigate the effects of QCLG on HE mice and explore the correlation between changes in gut microbiota and metabolites and HE.

## Materials and methods

### Reagents

QCLG was obtained from Beijing Tongrentang Drugstore. The QCLG comprised 5 Chinese medicinal materials, including *Rheum palmatum L.* [Polygonaceous; *Rhea Radix Et Rhizomes*.] *Rehmannia Radix* [Scrophulariaceae; *Rehmannia glutinosa Libosch*.] *Magnoliae Officinalis Cortex* [Magnoliaceae; Magnolia officinalis *Read. et Wils.*] *Aurantii Fructus* [Rutaceae; *Citrus aurantium L.*] *Taraxaci Herba* [Asteraceae; *Taraxacum mongolicum Hand-Mazz.*] The five raw botanical drugs were combined in a ratio of 2:1:1: 1:1 and subjected to two rounds of boiling with 10-fold deionized water (ddH_2_O, 124 w/v) for 1 hour each. Then, it is filtered to obtain the filtrate and stored in aliquots at 10 mL 125 at 4 °C before use. Thioacetamide (TAA) was obtained from Sigma-Aldrich (St. Louis, USA). The same batch of QCLG was used throughout the experiment and was not mixed with other products.

### Animals

Thirty male C57BL/6 mice (Beijing HFK Bioscience Co., Ltd.) weighing 20-25 g and specifically pathogen-free were used.

### Experimental design

30 mice were divided into 5 groups of 6 mice each. These five groups included normal control (NC), thioacetamide (TAA), treatment, lactulose, and QCLG groups. NC group was gavage with normal saline and injected intraperitoneally. The TAA group was gavage with normal saline and injected intraperitoneally with TAA. The Lactulose group was gavage with lactulose and injected intraperitoneally with TAA. The treatment group was gavaged with QCLG and injected intraperitoneally with TAA. The QCLG group was gavaged with QCLG and injected intraperitoneally with normal saline. The QCLG group was used to evaluate the effects of QCLG on normal mice. The feeding conditions were as follows: temperature 20~25°C, humidity 40%~60%, 12 hours of light per day, free access to food and water, and standard feed. All rats were adaptively reared for 1 week before experiments were conducted. TAA is considered an alternative drug in the guidelines for modeling HE (DeMorrow et al., 2021). Given the absence of a specified dose in the guidelines, we injected 150 mg/kg intraperitoneally for two consecutive days in this experiment following multiple screenings. Following a week of acclimatization feeding, 5 groups were gavage and given the drug at the same time. The treatment and QCLG group were given QCLG (6.725g/kg) for 7 days, the lactulose group was gavage with lactulose (167 mg/kg) for 7 days, and the other two groups (TAA group and NC group) were gavage with an equal amount of normal saline for 7 days. 24 hours after the intragastric administration, mice in the TAA group, treatment group, and lactulose group were intraperitoneally injected with TAA (150 mg/kg). The NC group and QCLG group were intraperitoneally injected with an equal volume of normal saline. After the final TAA injection, mice were anesthetized with ether 24 hours later, and the ether concentration was maintained at 2%-4%. Blood samples were collected from the retro-orbital venous plexus and underwent a 10-minute centrifugation at 1,800 g at 4°C to obtain serum for measuring blood ammonia, ALT, AST, and inflammatory factors. The mice were euthanized by cervical dislocation. Partial liver tissues were fixed with 4% paraformaldehyde for morphological analysis, and brain tissues were fixed in a 3% glutaraldehyde solution. Additionally, some brain tissues were frozen rapidly in liquid nitrogen for qPCR and immunohistochemical analysis. Fecal samples were rapidly frozen in liquid nitrogen for 16S rRNA and metabolomics analysis. All tissues were adequately frozen during the experiment. Organ coefficients were calculated according to the following standard:


Relative organ weight=[organ weight(g)/body weight (g)]×100%

All procedures were performed by the Guide for the Care and Use of Laboratory Animals established by the Beijing Municipal Ethics Committee. Animal experiments were approved by the Animal Welfare Committee of Capital Medical University (Approval Number: AEEI-2022-228).

### Open field tests

An open-field experiment is a method to evaluate the autonomous behavior, inquiry behavior, and tension of experimental animals in a new environment. It’s often used to detect anxiety, exploratory behavior, and exercise ability in mice. In this study, the behavior ability of mice was evaluated by an open-field experiment. Open field tests were performed on four groups (NC, TAA, treatment, and lactulose group) 12 hours after the last TAA injection. The open field apparatus, measuring 50 cm x 50 cm x 45 cm, included an image capture system and operational analysis tools. It was placed in a well-lit, noise-free environment to test up to four mice simultaneously. Mice were introduced one hour before the experiment, ensuring appropriate lighting and a calm setting. The experiment began by placing mice in the center of the square arena for five minutes of unrestricted exploration, recorded by an automated video tracking system. After each experiment, the arena floor was cleaned to prevent potential chemical interference from urine or feces. The assessment included measuring both distances traveled and average speed.

### Assessment of brain function

The assessment of brain function consists of 10 evaluation items, which can comprehensively evaluate the behavioral ability of mice. the higher the score, the worse the behavioral ability. Following the last TAA injection, brain function in four groups (NC, TAA, QCLG, treatment, and lactulose) was assessed 12 hours later. The evaluation utilized a 10-point system based on the method outlined by Chen et al (Avraham et al., 2011), covering criteria such as escaping from a circular ring, foraging behavior, corneal reflex, straight-line walking, startle reflex, grasping reflex, righting reflex, walking on a balance beam, placement reflex, and climbing behavior. Abnormal reflexes or behaviors scored 1 point, while normal behavior scored 0. Three individuals independently scored the assessments without communication to avoid bias. The equipment was cleaned after each experiment to prevent urine, feces, or odor interference.

### Serum biochemical analyses

The guidelines for animal models of hepatic encephalopathy point out that the animal model of hepatic encephalopathy is based on the presence of liver injury or failure and abnormal blood ammonia. Serum was used to detect ALT, AST, and blood ammonia levels in mice, and to evaluate liver injury, and blood ammonia levels. Serum ALT and AST were quantified utilizing Chemray 800 and Rayto, along with a fully automated Chinese biochemical analyzer. Blood ammonia levels were determined using the G0436W blood ammonia assay kit of Grace Biotechnology. All protocols were executed as per the provided user manual.

### Immunohistochemistry of glial fibrillary acid protein, Ib1 and γ-aminobutyric acid in brain tissues

Immunohistochemistry was used to detect the expression of microglia, astrocytes, and GABA in brain tissue. The above indicators were used to assess the degree of neuroinflammation in mice with hepatic encephalopathy. Tissue sections were deparaffinized and then incubated overnight at 4°C with anti-Ib1 mouse monoclonal antibody (Servicebio GB12105), anti-γ-aminobutyric acid (anti-GABA) A Receptor beta2/GABRB2 Rabbit polyclonal antibody (Servicebio GB114791), and anti-glial fibrillary acid protein (anti-GFAP) Rabbit polyclonal antibody (Servicebio GB11096) in a wet box. Subsequently, sections were washed and incubated for 50 minutes at room temperature with horseradish peroxidase (HRP)-labeled goat anti-mouse antibody (Servicebio GB23301) and HRP-labeled goat anti-rabbit immunoglobulin G (IgG, Servicebio GB23204). After additional washing, the tissue sections were developed with Diaminobenzidine (DAB), counterstained with hematoxylin, and examined under a bright-field microscope.

### Histopathological examination of the liver

Liver histopathology to assess the extent of liver damage. Liver and brain tissue samples were obtained from each mouse group. Liver tissues were fixed in a 4% formaldehyde solution, while brain tissues were fixed in a 3% glutaraldehyde solution. The severity of liver damage was assessed using the histological activity index (HAI) score. The prefrontal cortex was isolated from brain tissues, and the extent of brain lesions was determined by examining neuronal cell bodies and synapses.

### 16S rRNA gene sequencing

In this study 16S rRNA gene sequencing was used to investigate the effect of QCLG on the Intestinal microflora of mice with HE. Genomic deoxyribonucleic acid (DNA) was extracted from fecal intestinal microbiota using the PF Mag-Bind Stool DNA Kit (Omega Bio-Tek, USA). DNA concentration, integrity, and quality were assessed using NanoDrop2000 and 1% agarose gel electrophoresis. The V3-V4 region of the 16S rRNA gene was amplified with 338F upstream and 806R downstream primers (ACTCCTACGGGAGGCAGCAG and GGACTACHVGGGTWTCTAAT). PCR products were purified using a PCR clean-up kit. Subsequently, libraries were constructed using the NEXTFLEX Rapid DNA-Seq Kit and sequenced with Illumina PE300 (Illumina, USA).

### Metabolite analysis

Metabolite analysis was used to examine the effects of QCLG on metabolites in mice with hepatic encephalopathy. In a 2 mL centrifuge tube with a 50 mg fecal sample and a 6 mm diameter grinding bead, 400 μL of extraction solution (methanol to water ratio 4:1, v/v) with 0.02 mg/mL of internal standard (L-2-chlorophenylalanine) was used. LC-MS/MS analysis was performed on a Thermo Fisher Scientific UHPLC-Q Exactive HF-X system provided by Shanghai Meiji Biomedical Technology Co., Ltd., using a Thermo UHPLC-Q Exactive HF-X system with an ACQUITY HSS T3 column (100 mm × 2.1 mm i.d., 1.8 μm; Waters, USA) at Majorbio Bio-Pharm Technology Co. Ltd. (Shanghai, China). Progenesis QI software preprocessed LC/MS raw data (Waters Corporation, Milford, USA), excluding internal standard peaks and known false positives. Metabolite identification uses databases like the Human Metabolome Database (HMDB, http://www.hmdb.ca/) and Metlin (https://metlin.scripps.edu/).

### Enzyme-linked immunosorbent assay

Enzyme-linked immunosorbent assay was used to detect inflammatory factors in mouse serum and to evaluate the effect of QCLG on the inflammatory level in mice with HE. Enzyme-linked immunosorbent assay (ELISA) kits (Servicebio 88-7013-88 for Interleukin-1β (IL-1β), Servicebio GEM0001-96T for interleukin-6 (IL-6), and ServicebioGEM0004-96T for tumor necrosis factor-α (TNF-α) were utilized for detection. After that, 100 µL of coating buffer was added to each well of ™ Costar™ 9018 ELISA plates, which were sealed at 4°C overnight. After the washing buffer was used for washing, any remaining liquid was removed with absorbent paper, and the plates were sealed with 200 µL of 1X ELISA dilution buffer. After one hour of incubation at room temperature and washing, a standard curve was generated. Next, 100 µL of the sample was introduced into each well, and 100 µL of 1X ELISA dilution buffer was introduced into blank wells, which were sealed at 4°C overnight. By the same protocol, antibodies and Streptavidin-HRP were prepared. In addition, 100 µg per well was added to all wells, which were sealed for one hour at room temperature. Subsequently, each well was added with 100 µg of 1X Tetramethyl Benzidine (TMB) Solution at room temperature and underwent 15-minute incubation before the addition of the stop solution. The enzyme immunoassay was conducted at an absorbance of 450 nm, and the data were analyzed.

### Real-time quantitative PCR

Real-Time Quantitative PCR was used to detect the levels of inflammatory factors in mouse brain tissue and evaluate the effect of QCLG on neuroinflammation. Total RNA was isolated from the liver using TRIzol reagent (Catalog No. 15596018; ThermoFisher) and then transcribed to cDNA using a Strand cDNA Synthesis Kit (Catalog No. 6210A; Takara). Real-time quantitative PCR (RT-qPCR) was performed with LightCycler 480 and technical triplicates using TB Green reagent (Catalog No. RR420A; Takara). The expression levels were calculated with the 2^(-ΔΔCT)^ method, and the Cycle threshold (CT) values were normalized using GAPDH as a reference gene. The target genes were Thbs1 and Osgin1 (**Table 1**).

**Table 1:** Sequences of primers used in real-time polymerase chain reactions.

Genes	Sequences of primer	Annealing Tm (°C)
GAPDH Forward primer GAPDH Reverse primer IL-1β Forward primer IL-1β Reverse primer IL-6 Forward primer IL-6 Reverse primer TNF-α Forward primer TNF-α Reverse primer	5'-CAGTGGCAAAGTGGAGATTGTTG-3' 3'-CTCGCTCCTGGAAGATGGTGAT-5' 5'-TTCAGGCAGGCAGTATCACTC-3' 3'-GAAGGTCCACGGGAAAGACAC-5' 5'-CTGCAAGAGACTTCCATCCAG-3' 3'-AGTGGTATAGACAGGTCTGTTGG-5' 5'-CAGGCGGTGCCTATGTCTC-3' 3'-CGATCACCCCGAAGTTCAGTAG-5'	59.22 59.22 59.22 59.22 59.22 59.22 59.22 59.22

### Statistical analysis

SPSS and GraphPad were used for statistical analysis and plotting, respectively. Data were presented as mean ± SEM. T-tests or Wilcoxon rank-sum tests were used for between-group comparisons and one-way ANOVA with Bonferroni correction for multiple groups. Statistical significance was set at p < 0.05. PLS-DA with VIP > 1 and P < 0.05 selected metabolites and pathway analysis utilized the KEGG website (http://www.genome.jp/kegg/).

## Result

### Qingchang Ligan formula can improve the effects of TAA-induced HE on mice’s body and organ weights

**Figure 1:**
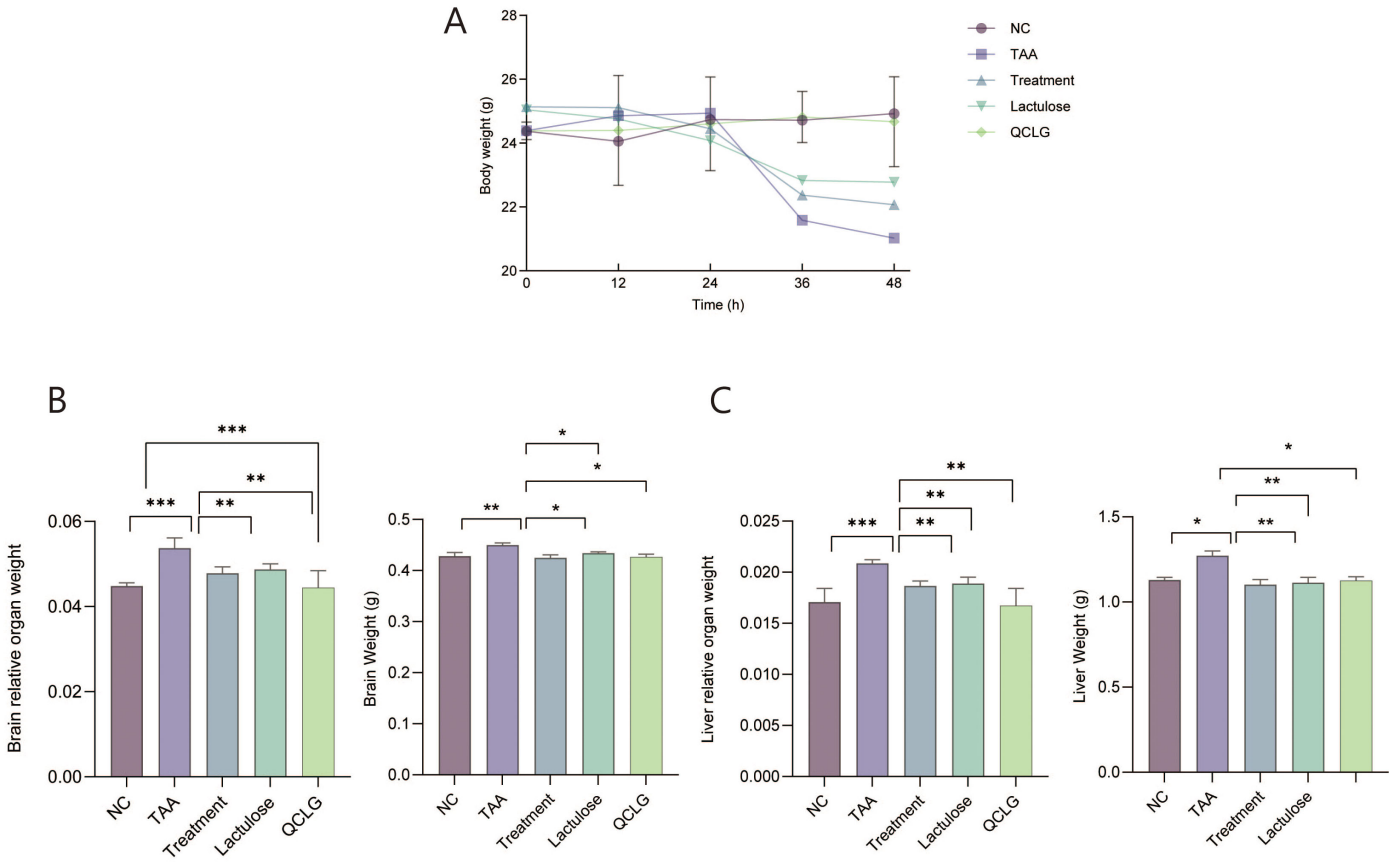
Qingchang Ligan Formula (QCLG) improves the effects of TAA-induced HE on body and organ weights. **(A)** Body weight was obtained 48 hours after an intraperitoneal injection of TAA. **(B)** Brain weight and Relative organ weight of the brain. **(C)** liver weight and Relative organ weight of the liver. NC, normal control group; TAA, thioacetamide model group; treatment, treatment group; lactulose, lactulose group; QCLG, Qingchang Ligan Formula group. Data were presented as mean ± SEM. (n = 5) *P < 0.05, **P < 0.01 and ***P < 0.001.

Mice in the TAA group exhibited a significant reduction in body weight compared to the NC group, while those in the treatment and lactulose groups showed a significant increase (**Figure 1A**). The liver and brain relative weights were notably higher in the TAA group than in the NC group. In contrast, the treatment and lactulose groups displayed a significant decrease in relative weight compared to the TAA group (**Figures 1B, C**).

### QCLG can improve the behavioral abnormalities in HE mice

**Figure 2:**
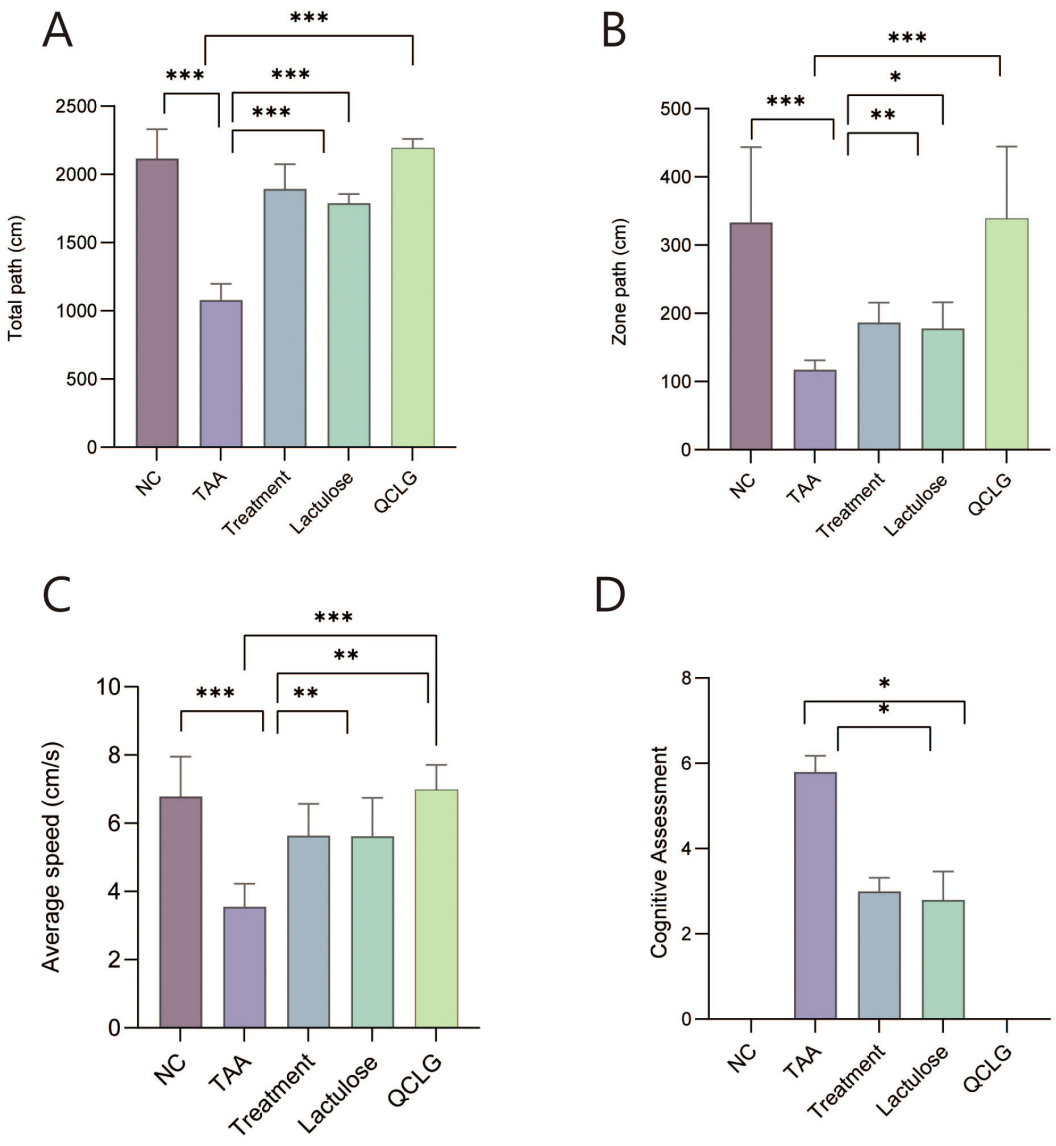
Effects of QCLG on behavioral tests: Open field tests and cognitive assessments **(A)** Total path, **(B)** Zone path, **(C)** Average speed, **(D)** Cognitive assessments, NC, normal control group; TAA, thioacetamide model group; treatment, treatment group; lactulose, lactulose group; QCLG, Qingchang Ligan Formula group. Data were presented as mean ± SEM. (n = 5) *P < 0.05, **P < 0.01 and ***P < 0.001.

Individual open-field tests and cognitive assessments were conducted on each mouse group to assess behavioral effects. The TAA group significantly reduced total distance traveled, zone-specific distance, and average speed compared to the NC group in open field tests (**Figures 2A–C**). Conversely, lactulose and treatment groups showed a significant increase in these parameters compared to the TAA group. In cognitive assessments, the TAA group exhibited a significant increase in scores compared to the NC group. In contrast, lactulose and treatment groups significantly decreased scores compared to the TAA group. There was no significant difference between QCLG and NC groups (**Figure 2D**).

### QCLG can protect against TAA-induced liver damage and peripheral inflammation

**Figure 3:**
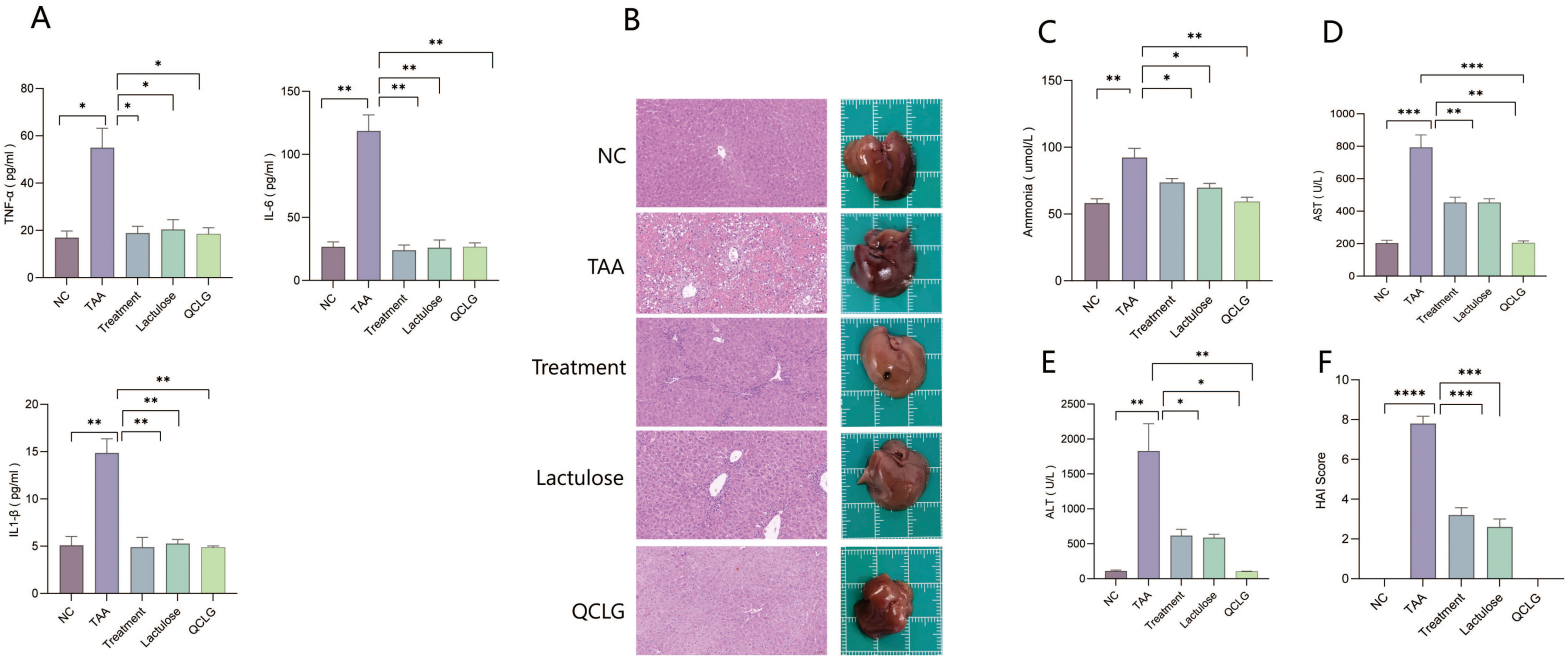
QCLG alleviates TAA-induced liver injury and the levels of ammonia, TNF-α, IL-6 and IL-1β in the blood **(A)** The levels of TNF-α, IL-6 and IL-1β in the blood, **(B)** Representative liver sections from each group, Liver tissue hemorrhage and inflammatory infiltration (red border) **(C)** Plasma ammonia was confirmed 48 hours after an intraperitoneal injection of TAA, **(D)** Plasma AST levels were confirmed 48 hours after an intraperitoneal injection of TAA, **(E)** Plasma ALT levels were confirmed 48 hours after an intraperitoneal injection of TAA, **(F)** HAI score of the liver. NC, normal control group; TAA, thioacetamide model group; treatment, treatment group; lactulose, lactulose group; QCLG, Qingchang Ligan Formula group. Data were presented as mean ± SEM. (n = 5) *P < 0.05, **P < 0.01 and ***P < 0.001.

Peripheral inflammation can worsen liver damage, influencing the development of HE (DeMorrow et al., 2021). Serum levels of TNF-α, IL-1β, and IL-6 were assessed in mice. The TAA group showed significantly elevated TNF-α, IL-1β, and IL-6 levels compared to the NC group. In contrast, the treatment and lactulose groups exhibited notable reductions in these inflammatory factors compared to TAA. There was no significant difference between QCLG and NC groups (**Figure 3A**). Image analysis revealed increased liver tissue bleeding and irregular surfaces in the TAA group compared to the NC group, while the treatment and lactulose groups displayed smoother liver surfaces. There was no significant difference in the surface between the NC group and the QCLG group. (**Figure 3B**).

ALT, AST, and blood ammonia levels, crucial indicators for assessing HE severity and liver injury biomarkers, were assessed. The TAA group showed a significant increase in ALT, AST, and blood ammonia levels compared to the NC group. Conversely, the treatment and lactulose groups exhibited a considerable reduction in ALT, AST, and blood ammonia levels relative to TAA (**Figures 3C–E**). Histopathological analysis revealed heightened liver cell necrosis, cell swelling, inflammatory cell infiltration, and extensive bleeding in the TAA group compared to the NC group. Treatment and lactulose groups demonstrated a marked decrease in these pathological features relative to TAA. There was no significant difference between QCLG group and NC group in the above indexes. (**Figures 3B, F**).

### Treatment with QCLG can restore TAA-induced neuroinflammation

**Figure 4:**
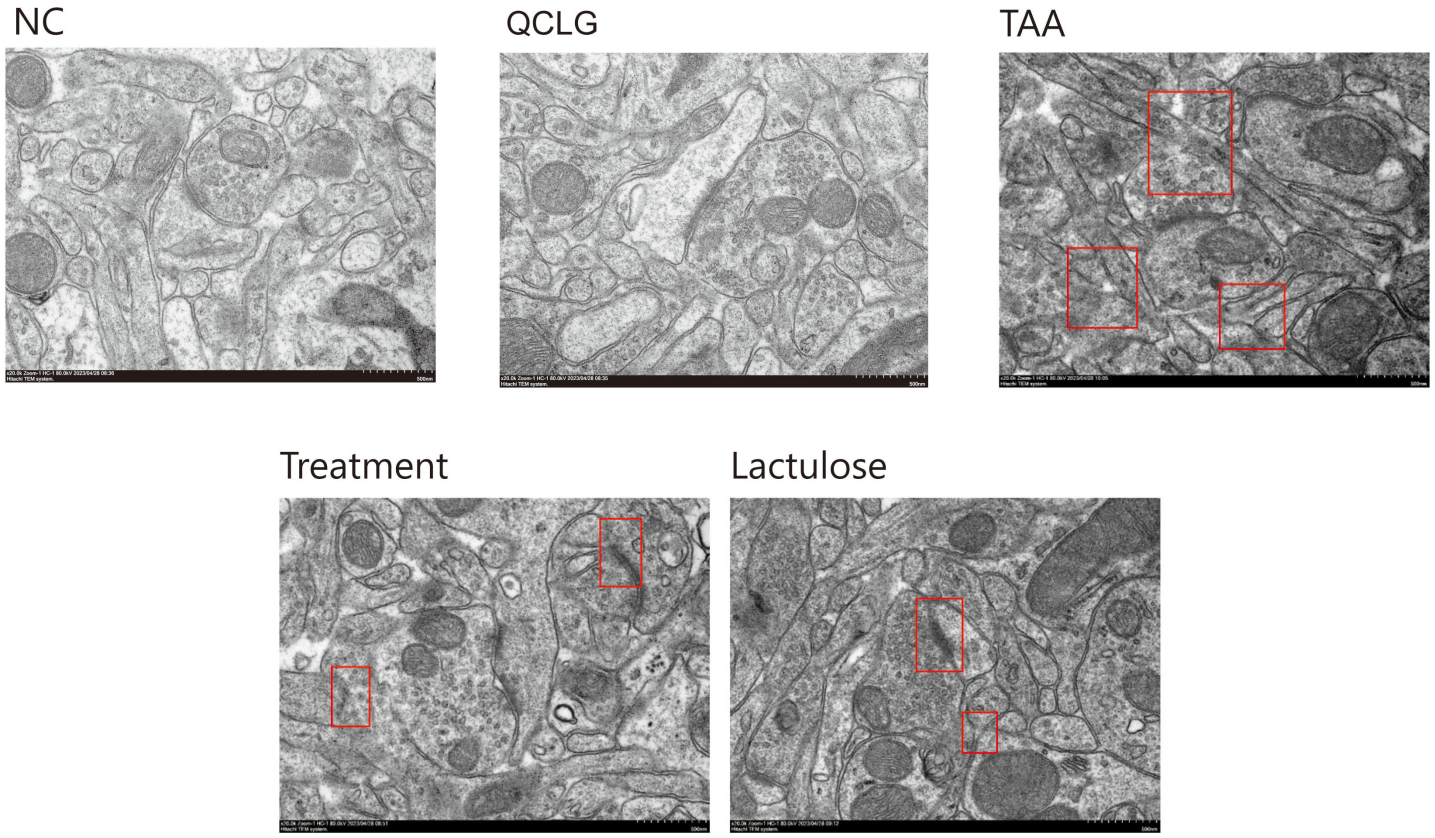
QCLG alleviates TAA-induced neuronal damage. NC, normal control group; TAA, thioacetamide model group; treatment, treatment group; lactulose, lactulose group; QCLG, Qingchang Ligan Formula group. Synaptic structure (red border).

The severity of brain injury and neuroinflammation significantly influences the progression of HE. Neuronal soma and synapse changes serve as indicators for assessing brain lesions. Transmission electron microscopy (TEM) analyzed QCLG’s influence on neurons and examined brain tissue neuronal structure (**Figure 4**). In the TAA group, mice showed neuronal swelling and partial dendritic loss compared to the NC group. However, the treatment group demonstrated significant improvement. In the prefrontal cortex and striatal synaptic structures (**Figure 4**), NC group synaptic structures were intact, with several synaptic vesicles, clear synaptic cleft, average C width, and standard postsynaptic density thickness. In TAA group mice, synaptic structures were unclear; synapses showed varying degrees of swelling, reduced synaptic vesicles, inconsistent synaptic cleft width, and thinning postsynaptic density. The treatment group’s synaptic structures showed significant relief compared to the TAA group, with increased synaptic vesicles and some improvement in synaptic cleft width.

**Figure 5:**
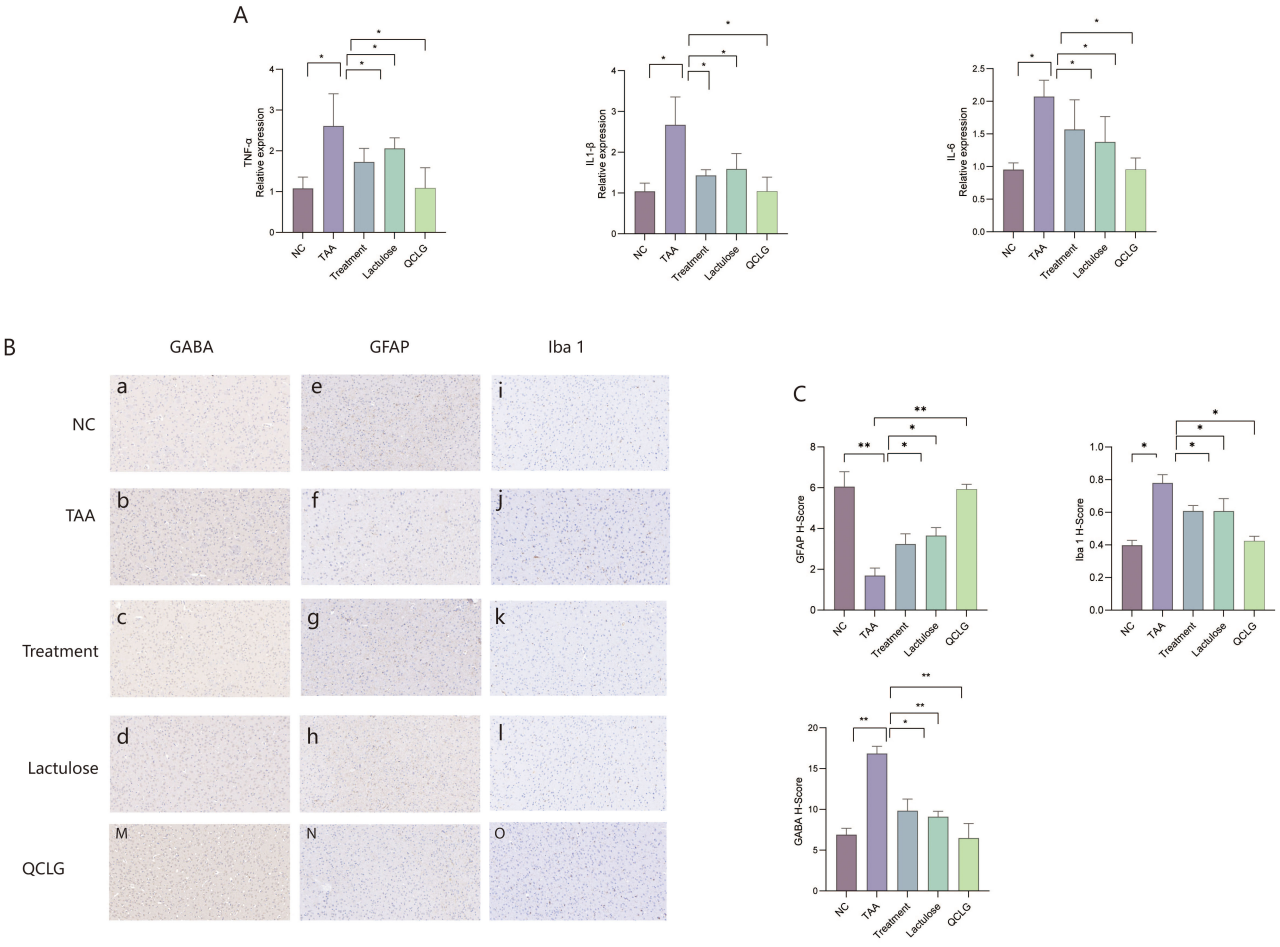
QCLG reduces neuroinflammation. **(A)** QCLG alleviates TNF-α, IL-6 and IL-1β in the brain. **(B)** Immunohistochemical evaluation of GFAP, GABA and Iba1 (40x zoom level). **(C)** Image analysis of GABA, Iba1, and GABA protein expression. NC, normal control group; TAA, thioacetamide model group; treatment, treatment group; lactulose, lactulose group; QCLG, Qingchang Ligan Formula group. Data were expressed as mean ± SEM (n = 5). *P < 0.05, **P < 0.01.

In assessments related to neuroinflammation, the results revealed a significant increase in TNF-α, IL-1β, and IL-6 levels in the brain tissue of the TAA group compared to the NC group. Conversely, the lactulose and treatment groups substantially decreased these three inflammatory factors compared to the TAA groups (**Figure 5A**). Meanwhile, markers of astrocyte activation (GFAP), microglia (ionized calcium-binding adapter molecule 1, iba1), and GABA expression in brain tissue were assessed. Astrocyte staining in the TAA group appeared lighter and had a significantly lower H-Score than that in the NC group. About iba1, microglia staining in the TAA group was darker and had a significantly higher H-Score than in the normal group. Regarding γ-aminobutyric acid (GABA), GABA staining in the TAA group was darker and had a significantly higher H-Score than in the NC group. There was no significant difference between QCLG and NC groups (**Figures 5B, C**).

### QCLG can alter gut microbiota structure

**Figure 6:**
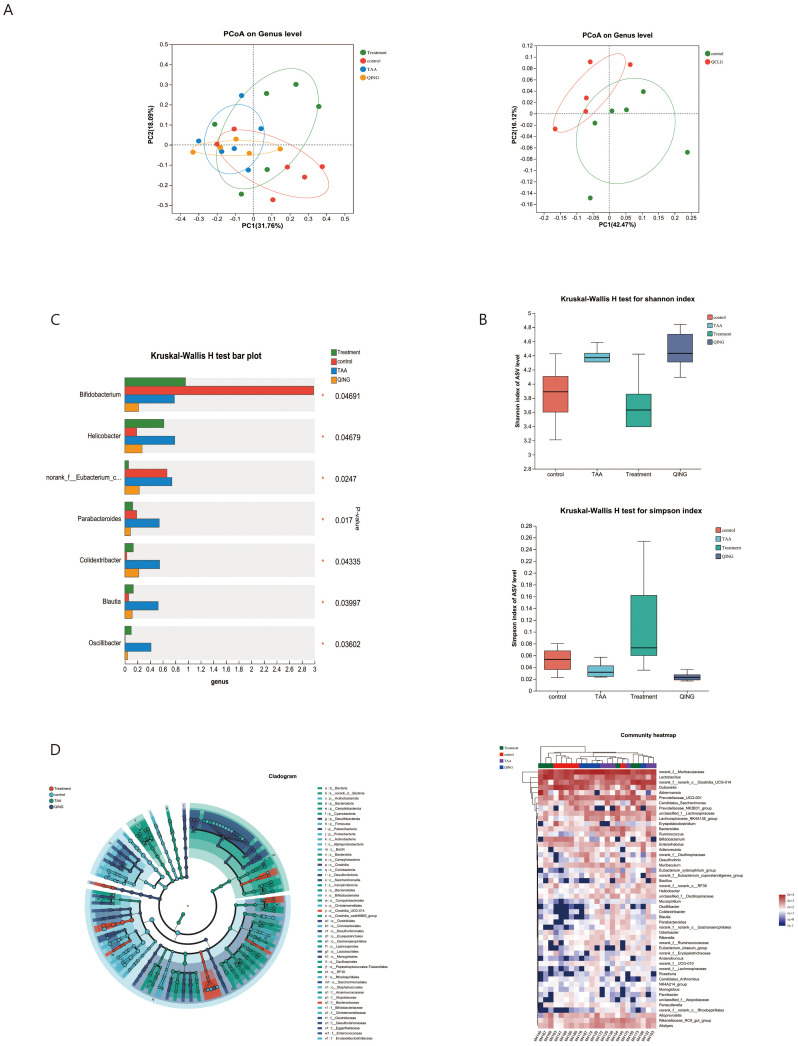
Effects of QCLG on the composition and structure of the gut microbiota (n = 5-6). **(A)** β diversity was up to the principal based on unweighted-unifrac distance. **(B)** α diversity. **(C)** Alterations in the relative abundances of genus-level bacterial taxa in treatment, QCLG, NC and TAA groups (*P < 0.05, one-way ANOVA). **(D)** Graphical phylogenetic analysis of changes in the gut microbiota. Heatmap of the relationships between microbiota and other experimental results. NC, normal control group; TAA, thioacetamide model group; Treatment, treatment group; QCLG, Qingchang Ligan Formula group.

A body of research has emphasized the two-way association of the brain with the gut, where changes in the gut microbiota are linked to inflammation (Bajaj et al., 2015). To elucidate the molecular mechanisms underlying QCLG treatment of HE, we conducted 16S rRNA analysis on mouse feces and performed β-diversity analysis to assess differences between groups. Principal coordinate analysis (PCoA) based on Unweighted-unifrac dissimilarity demonstrated separation between the control group and both model and QCLG-treated groups. Notably, the TAA group exhibited distinct differences in gut microbiota structure compared to the QCLG-treated one. To confirm whether these changes were induced by QCLG, the QCLG group was also compared with the control one, which revealed a divergence (explaining 42.47% of the variance), indicating significant alterations in the core microbiota after treatment (**Figure 6A**). The diversity of the microbial community was assessed using diversity indices (Shannon and Simpson indices) (**Figure 6B**). It was observed that the fecal samples of TAA-treated mice showed a remarkable increase in microbial diversity compared to those of NC mice. In contrast, QCLG intervention led to a marked reduction in gut microbial diversity. The QCLG group exhibited a substantial increase in gut microbial diversity. The analysis of the microbiota composition of mouse feces revealed notable differences. The TAA group demonstrated a significant increase in the abundance of *Parabacteroides*, *norank-f–Eubacterium-coprostanoligenes-group* (*P* = 0.013), *Oscillibacter* (*P* = 0.028), *Blautia* (*P* = 0.029), *Colidextribacter* (*P* = 0.021) and *Helicobacter* (*P* = 0.044) compared with the normal one. However, the abundance of *Bifidobacterium* (*P* = 0.039) notably decreased. Conversely, these microorganisms displayed a marked decrease in the QCLG-treated group compared with the TAA one, with a significant increase in *Bifidobacterium* (**Figure 6C**). Furthermore, the analysis revealed differences in the abundance of particular taxa, as shown in the heatmap and dendrogram. The analysis indicated that HE was associated with higher levels of *Desulfovibrio* (*P* = 0.007), *Helicobacter* (*P* = 0.044), *Oscillibacter* (*P* = 0.028), *Colidextribacter* (*P* = 0.021) and *Rikenella* (*P* = 0.025). However, these levels significantly reduced QCLG-treated mice (**Figure 6D**).

### QCLG can alter relevant metabolites in the intestines

**Figure 7:**
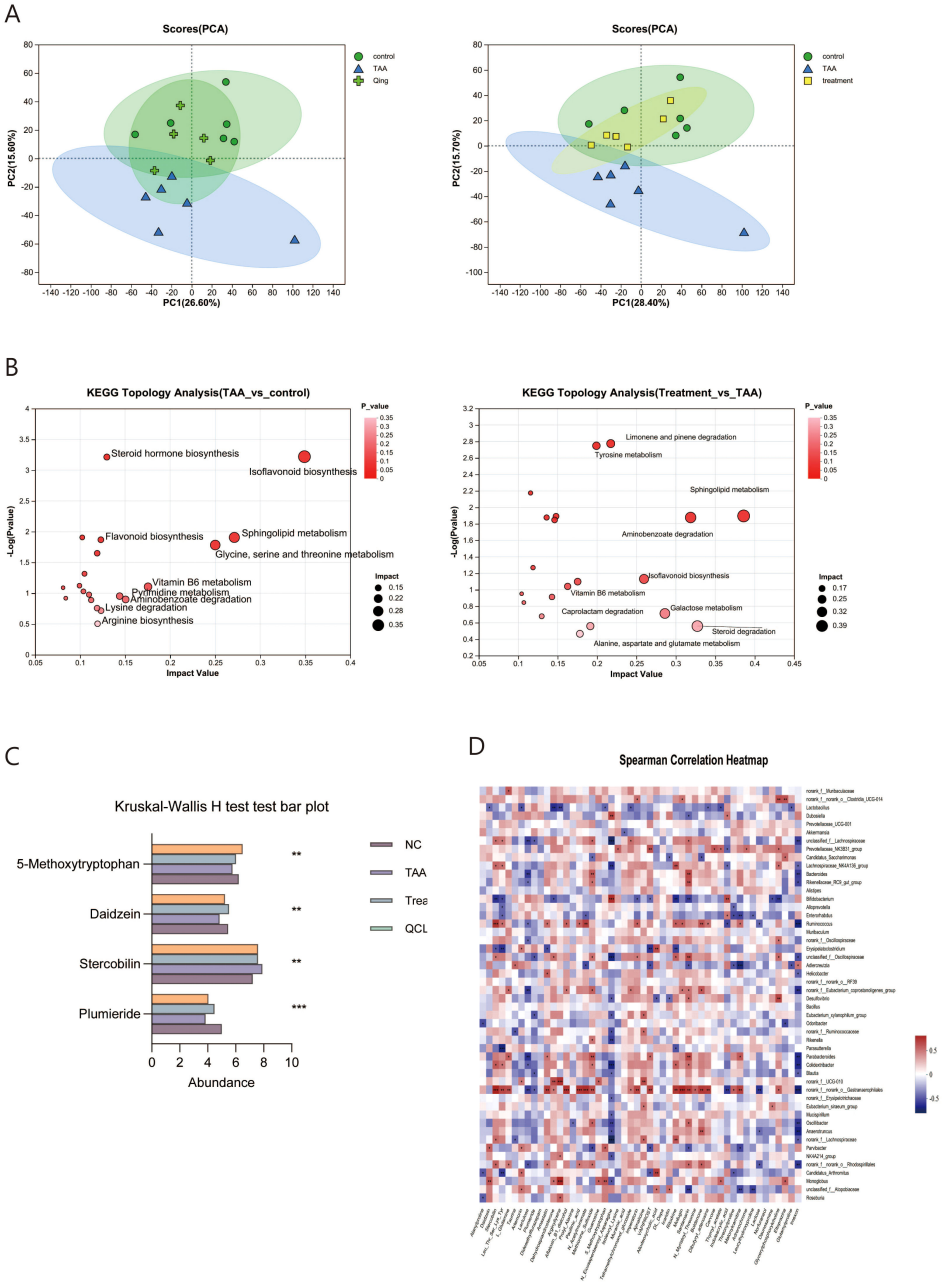
QCLG can alter relevant metabolites in the intestines (n = 5-6). **(A)** PCA (PC1 = 26.6%, PC1 = 28.40%) **(B)** Meaningful metabolic routes in comparing NC and TAA groups, TAA and treatment groups. **(C)** Based on *VIP* > 1 and *P* < 0.05, false discovery rate (FDR) < 0.05 served as a filter for differential metabolites between NC and TAA groups. QCLG treatment contributed to a significant improvement in metabolic disorders. **(D)** Heatmap of the association between the changed microbial community and greatly changed metabolites. NC, normal control group; TAA, thioacetamide model group; treatment, treatment group; lactulose, lactulose group; QCLG, Qingchang Ligan Formula groups. *P < 0.05, **P < 0.01.

The complex interactions between the host and the intestinal microbiota are closely related to the host-microbe metabolic axis. To verify the impact of QCLG, we conducted untargeted metabolomic studies on stool samples using liquid chromatography-mass spectrometry (LC-MS). In negative and positive modes, 589 and 796 metabolites were identified in fecal samples. In negative and positive modes, 589 and 796 metabolites were identified in fecal samples. To determine the specific effects of QCLG on metabolites, we conducted PCA on the TAA, QCLG, and NC groups. PCA (26.60%) demonstrated clustering of metabolites in the NC group and QCLG group, with the TAA group showing significant differences compared with the other two groups (**Figure 7A**).

In analyzing the metabolites across the three groups, we found 946 distinct metabolites. KEGG pathway enrichment analysis revealed that the Model group had significant pathways compared to the normal group, including Isoflavonoid biosynthesis, steroid hormone biosynthesis, flavonoid biosynthesis, sphingolipid metabolism, glycine, serine and threonine metabolism, Vitamin B6 metabolism, pyrimidine metabolism, aminobenzoate degradation, lysine degradation, and arginine biosynthesis. Notably, QCLG treatment differed from the TAA group in pathways such as sphingolipid metabolism, aminobenzoate degradation, caprolactam degradation, galactose metabolism, steroid degradation, and alanine aspartate glutamate metabolism. This suggests that QCLG may mitigate some effects of TAA through these metabolic pathways (**Figure 7B**).

Subsequently, to identify potential biomarkers of QCLG treatment efficacy, we employed Student’s t-test to compare the metabolic alterations in HE across the three groups. We identified 307 metabolites that significantly changed between the NC group and the TAA group (VIP>1, P<0.05, FDR<0.05). 179 metabolites gradually returned to normal levels following QCLG treatment (P<0.05). Within this group, the QCLG treatment upregulated 106 metabolites that were diminished by TAA and down-regulated 73 other metabolites, including 5-Methoxytryptophan, Daidzein, Stercobilin, and Plumieride (PLU), bringing their levels closer to those of the NC group (**Figure 7C**).

Additionally, our Spearman correlation analysis of the microbiota and metabolites revealed correlations between the top 50 most abundant intestinal microbial communities and 50 differentially altered fecal metabolites. Stercobilin, Leu-Thr-Ser-Lys-Tyr, and Amastatin exhibited positive correlations with *Blautia*, whereas 5-methoxy tryptophan and Plumieride showed negative correlations with *Blautia*. Furthermore, Stercobilin, Leu-Thr-Ser-Lys-Tyr, Dehydroepiandrosterone, Muramic-acid, Imperatorin, DL-dopa, Ritodrine, Lactose, and Danunosamine showed a negative correlation with *Bifidobacterium*. At the same time, *Bifidobacterium* exhibited a positive correlation with Indoleacrylic-acid, N-Eicosapentaenoyl-Asparagine, Daidzein, 5-methoxy tryptophan, and Plumieride. Additionally, *Oscillibacter* exhibited positive correlations with Daidzein, Stercobilin, Methionine-Sulfoxide, Santamaria, Lumichrome, Prolyl-Alanine, N-Eicosapentaenoyl-Asparagine, Imexon, 5-methoxy tryptophan, and Plumieride. In contrast, *Oscillibacter* was negatively correlated with Lumichrome, Prolyl-Alanine, N-Eicosapentaenoyl-Asparagine, Imexon, 5-methoxy tryptophan, and Plumieride (**Figures 7D**).

## Discussion

We created a mouse model of HE through intraperitoneal injection of TAA to assess the therapeutic impact of QCLG and delve into its potential mechanism. TAA serves as a preclinical model for HE established following animal modeling guidelines, demonstrating effective replication of human acute liver disease. This method is widely accepted for inducing HE (DeMorrow et al., 2021). The experimental results unequivocally demonstrate the efficacy of this method in inducing characteristic HE symptoms. In our primary study, QCLG markedly enhanced behavioral and cognitive functions affected by HE, mitigated brain inflammation, rectified microbial imbalances, and improved metabolic status. We summarized the outcomes of 16SrRNA gene sequencing and metabolomic analysis, exploring the impact of QCLG on HE by scrutinizing the interplay between intestinal bacteria and metabolic biomarkers.

The neuropathology of HE involves astrocyte reduction, microglia activation, and neuroinflammation (Butterworth, 2019; Hsu et al., 2021). Immunohistochemistry analysis revealed significant differences between the NC and TAA groups in astrocyte, microglia, and GABA expression. The TAA group showed increased microglia and GABA expression and reduced astrocytes. Astrocyte dysfunction disrupts the brain neurotransmission system, which subsequently causes a cascade of neuronal injuries and ultimately results in neurocognitive deficits associated with HE (Butterworth, 2016). Microglia belong to the resident macrophage of the brain and play a pivotal role in innate immunity. Microglia activation results in chronic brain inflammation and an increase in proinflammatory cytokines such as TNF-α, IL-1β, and IL-6, which may be closely linked to the pathological features of HE (Hsu et al., 2021; Liu et al., 2021). Meanwhile, GABA is the brain’s primary inhibitory neurotransmitter, regulating emotions, memory, and appetite (Bäckström et al., 2021). Therefore, this suggests that QCLG can mitigate neuroinflammation in HE, thereby alleviating cognitive abnormalities.

The gut microbiota is a crucial neuroinflammation regulator in neurological diseases like HE. Recent advancements in metagenomics, Metatranscriptomics, and meta-proteomics have elucidated the functional interplay between the gut microbiota and central nervous system (CNS) function, known as the “gut-brain axis.” The gut microbiota is pivotal in numerous central nervous system diseases (Yu et al., 2021). Recent research indicates the involvement of the gut microbiota in modulating immune and inflammatory responses in acute and chronic neurological diseases (Li et al., 2021).

Our study shows that QCLG can alleviate this by regulating intestinal flora and metabolites. There were significant differences in the gut microbiota of HE mice compared with normal control mice. At HE, the relative abundance of *Bifidobacterium* decreased. Supplementation with *Bifidobacterium* alleviated cognitive deficits in mice and suppressed neuroinflammation and synaptic dysfunction (Zhu et al., 2023). At the same time, *Bifidobacterium* can also significantly regulate quinolinic acid (QUIN) levels in the brain, as well as glutamate (Glu) and GABA levels, thereby reducing the activity of microglia in the cerebellum (Kong et al., 2022). This means that QCLG can alleviate HE by regulating the abundance of beneficial bacteria. In the TAA group, the abundance of *Oscillibacter, Colidextribacter, Blautia*, and *Helicobacter* increased, and these significantly changed genera may be the signature bacteria of HE. Studies have shown that the abundance of *Oscillibacter* is relatively reduced after anti-inflammatory treatment in AD rats with neuroinflammation (Wang et al., 2022). At the same time, the reduction of *Oscillibacter* helps improve cognitive function and enhance learning and memory abilities after exercise (Zhou et al., 2021).

*Colidextribacter* is classified under the *Clostridiales cluster IV* and the *Clostridium cluster* Effect (Wang et al., 2022). Its involvement in raising cellular oxidative stress levels elevates serum inflammatory markers (Duan et al., 2021). The reduction in *Colidextribacter a*bundance may contribute to mitigating the impact of peripheral inflammation on neuroinflammation. The genus *Blautia* is classified within the family *Ruminococcaceae*, order *Clostridiales*, phylum *Firmicutes*, and class *Clostridia*. GABA is part of the *Blautia*-dependent arginine metabolism, closely linked to HE and Alzheimer’s disease (AD). Alterations in GABA levels can impact the susceptibility to mental disorders. Research indicates a robust association with arginine metabolism, potentially contributing to the pathogenesis of AD by modulating metabolites like GABA (Zhuang et al., 2020).

QCLG treatment normalized metabolite levels that differed significantly between the normal control and model groups. Upregulated metabolites in the QCLG group included Daidzein, a dietary metabolite with known anti-inflammatory properties (Das et al., 2018).. Daidzein has been shown to protect neurons by reducing neuronal apoptosis, enhancing neurite outgrowth, and promoting astrocytes’ production of neurotrophic factors, thereby preventing neuroinflammatory damage (Subedi et al., 2017).

As an anti-inflammatory endothelial factor, 5-methoxy tryptophan (5-MTP) safeguards the endothelial barrier, promotes endothelial repair, and inhibits the migration and proliferation of vascular smooth muscle cells via suppressing p38 mitogen-activated protein kinase (MAPK) activation (Wu et al., 2020). It is crucial in anti-inflammation, anti-cancer, and myocardial protection. PLU is a cyclic terpenoid compound extracted from willow flowers and exhibits anti-inflammatory, antidepressant, anxiolytic, and other effects (Dalmagro et al., 2020). Studies have shown that PLU can lower serum levels of ALT, AST, and alkaline phosphatase (ALP), thereby reducing liver damage (Singh et al., 2014).

In the down-regulated metabolites of the QCLG treatment group, Stercobilin and fecal pigments are demonstrated to trigger proinflammatory responses in the macrophage RAW264 cells of mice. This stimulation releases TNF-α, IL-1β, and other inflammatory factors, intensifying inflammation (Sanada et al., 2020; Dai et al., 2022).

Therefore, metabolites may collaboratively ameliorate HE through various direct and indirect pathways, such as inhibiting the secretion of inflammatory factors, promoting astrocytes to release neurotrophic factors, and mitigating liver damage.

Collectively, we speculate that QCLG can reduce neuroinflammation by regulating intestinal microbiota metabolism, thereby preventing HE.

Our study has limitations, including the need to investigate if the decoction of Chinese medicine alone or in combination alters the efficacy and composition of QCLG, a complex herbal formula. At the same time, we did not test the intestinal tissue. Changes in intestinal permeability will help us to explore the mechanism of hepatic encephalopathy more deeply. We will make up for this deficiency in the next step. Future research will explore the impact of changes in bacterial flora on intestinal inflammation, the influence of intestinal inflammation on HE progression, and the potential of QCLG for fecal microbiota transplantation therapy.

## Conclusion

Our study, for the first time, reveals the protective effect of QCLG treatment on a TAA-induced HE mouse model. Further mechanistic studies show that QCLG can ameliorate intestinal flora disorder and regulate metabolic abnormalities. Furthermore, we demonstrate the importance of microbiota dysbiosis in the pathogenesis of TAA-induced HE.

## Data Availability

The datasets presented in this study can be found in online repositories. The names of the repository/repositories and accession number(s) can be found in the article/supplementary material.
